# Comparison of body composition assessment by DXA and BIA according to the body mass index: A retrospective study on 3655 measures

**DOI:** 10.1371/journal.pone.0200465

**Published:** 2018-07-12

**Authors:** Najate Achamrah, Guillaume Colange, Julie Delay, Agnès Rimbert, Vanessa Folope, André Petit, Sébastien Grigioni, Pierre Déchelotte, Moïse Coëffier

**Affiliations:** 1 Nutrition Department, Rouen University Hospital Center, Rouen, France; 2 Normandie Univ, URN, INSERM UMR 1073, « Nutrition, Inflammation et dysfonction de l’axe Intestin-Cerveau », IRIB, Rouen, France; 3 Clinical Investigation Centre CIC 1404, INSERM and Rouen University Hospital, Rouen, France; University of Sydney, AUSTRALIA

## Abstract

**Background and aims:**

Body composition assessment is often used in clinical practice for nutritional evaluation and monitoring. The standard method, dual-energy X-ray absorptiometry (DXA), is hardly feasible in routine clinical practice contrary to Bioelectrical Impedance Analysis (BIA) method. We thus aimed to compare body composition assessment by DXA and BIA according to the body mass index (BMI) in a large cohort.

**Methods:**

Retrospectively, we analysed DXA and BIA measures in patients followed in a Nutrition Unit from 2010 to 2016. Body composition was assessed under standardized conditions in the morning, after a fasting period of 12 h, by DXA (Lunar Prodigy Advance) and BIA (Bodystat QuadScan 4000, Manufacturer’s equation). Bland-Altman test was performed for each class of BMI (kg/m^2^) and fat mass and fat free mass values were compared using Kruskal-Wallis test. Pearson correlations were also performed and the concordance coefficient of Lin was calculated.

**Results:**

Whatever the BMI, BIA and DXA methods reported higher concordance for values of FM than FFM. Body composition values were very closed for patients with BMI between 16 and 18,5 (difference < 1kg). For BMI > 18,5 and BMI < 40, BIA overestimated fat free mass from 3,38 to 8,28 kg, and underestimated fat mass from 2,51 to 5,67 kg compared with DXA method. For BMI ≥ 40, differences vary with BMI. For BMI < 16, BIA underestimated fat free mass by 2,25 kg, and overestimated fat mass by 2,57 kg. However, limits of agreement were very large either for FM and FFM values, irrespective of BMI.

**Conclusion:**

The small bias, particularly in patients with BMI between 16 and 18, suggests that BIA and DXA methods are interchangeable at a population level. However, concordance between BIA and DXA methods at the individual level is lacking, irrespective of BMI.

## Introduction

It is widely accepted that body composition can independently influence health [1–4]. Thus, body composition assessment is often used in clinical practice for nutritional evaluation and monitoring, such as in investigations of obesity and malnutrition, weight loss composition following bariatric surgery, sarcopenia in aging, osteopenia and osteoporosis. There are several available accurate techniques for the assessment of body composition in human [5].

Dual-energy X-ray absorptiometry (DXA) provides a rapid and non-invasive assessment of FM (fat mass), FFM (fat free mass) and bone mineral density, and is considered to be the reference method in clinical research [6]. Nevertheless, DXA requires specialised radiology equipment and is expensive, and thus hardly feasible in routine clinical practice. Inversely, Bioelectrical Impedance Analysis (BIA) method is commonly used for body composition assessment in clinical practice and research studies. Indeed, BIA is a simple, non-invasive, low-cost device which estimates the total body water (TBW) through the resistance of the body to a small alternating current [7]. Several BIA devices are available. Early systems used a single-frequency (SF-BIA) current and predictive equations involving the body’s resistance to current flow, and other variables such as weight, height and age [8]. However, BIA equations developed in a specific population are only generalizable to similar populations and caution is needed when applying to a population different from the validation sample, in order to avoid imprecise results and misinterpretation. More recently, multiple frequencies-BIA (MF-BIA) has been developed and allows prediction of (i) intracellular and extracellular water independently, and (ii) especially the phase angle which is known to decrease with age and height, and increase with greater FFM in men and women [9]. A low phase angle is associated with worse overall health outcomes [10–12]. Phase angle is calculated from arctangent of the reactance-to-resistance ratio, with the advantage of being independent of equations [10].

Limitations of BIA include assumptions involving a fixed hydration [13]. For instance, several factors limit the accuracy of BIA in patients with severe obesity: (i) many predictive equations has been developed in normal-weight subjects, (ii) body water distribution may be different in severe obesity state [14, 15]. Thus, BIA generally underestimated FM in patients with obesity [16, 17]. Although, Sartorio et al. reported accurate estimate of TBW in women with a wide range of body mass index (BMI) (19.1–48.2 kg/m) using BIA [18].

Comparison of body composition assessment by DXA and BIA according to the BMI has been poorly documented. Few studies have shown good concordance between the two methods [19, 20] while many others have not [16, 21–27]. These conflicting results may probably be due to some limiting factors including the use of different BIA devices (SF-BIA, MF-BIA) with different manufacturer equations, a small population size, and the differences in age, ethnicity and body weights in the sample studied. In our study, we aimed to compare body composition assessment by DXA and BIA according to the BMI in a large cohort of patients.

## Materials and methods

### Subjects

From 2010 to 2016, patients were included at the Department of Clinical Nutrition (University Medical Center, Rouen, France). Patients were included if they were aged above 18 years, without acute diseases, followed for malnutrition, obesity, or eating disorder. After an overnight fasting period of 12h, weight and height were measured by the same operator dressed light clothes without shoes. BMI was calculated as body weight (kg) divided by squared height (m^2^). The study was approved by the Local Ethics Committee for Non-Interventional Studies (CERNI, Comité d'Ethique pour la Recherche non interventionnelle) and all data were fully anonymized.

### Dual-energy X-ray absorptiometry (DXA)

DXA was performed on the whole body using a Lunar Prodigy Advance (General Electric Healthcare) without specific preparation. The assessment of QA and QC data for the DXA measurements was done every morning when patient assessment was planned and at least 3 days per week. The QA and QC data were sent each month to an independent security and control society for monitoring. Over the 6-year period, no deviation was observed and there was no firmware or software upgrades. The manufacturer controlled the DXA equipment at least one time per year. During measurement, all patients had their underwear on and no metal accessories worn. DXA uses an X-ray generating source, with two X-ray beams with different energy levels. Based on their X-ray attenuation properties, FFM (lean mass and bone mineral content) and FM were measured.

### Bioelectrical impedance analysis (BIA)

Body composition, FFM and FM, was assessed using multifrequency bioelectrical impedance analysis (BIA, Bodystat Quadscan 4000) as previously described [28], according to the manufacturer's recommendations. A calibration was done at least 2 times per year by using a manufacturer calibrator measuring impedance at each frequency. The Quadscan 4000 device records impedance at four frequencies (5, 50, 100 and 200 kHz), while only the 50 kHz impedance is used for the calculation of total body water, on which estimations for FFM are based using proprietary equations.

### Statistical analysis

The results of FFM and FM obtained by DXA and BIA (means ± sem) were compared using Bland-Altman test for the whole population and then, for each BMI class. Values of FFM and FM were also compared by Kruskal-Wallis test and Dunn’s multiple comparison tests. Pearson correlations were also performed and the concordance coefficient of Lin was calculated [29]. A difference with a p value < 0.05 was considered significant.

### Results

*Table 1* shows anthropometric data, body composition assessed by DXA and BIA in the included subjects (653 men and 3002 women) according to the BMI classes: BMI < 16; 16 ≤ BMI < 18.5; 18.5 ≤ BMI < 25; 25 ≤ BMI < 30; 30 ≤ BMI < 35; 35 ≤ BMI < 40 and BMI ≥ 40. Obese patients with BMI > 30 represented 74% of the population studied while patients with BMI < 18.5 represent 10%. Patients with BMI < 25 were younger than overweight and obese patients (p<0.05).

**Table 1:** Anthropometric data, body composition assessed by DXA and BIA.

	BMI < 16	16 ≤ BMI < 18.5	18.5 ≤ BMI < 25	25 ≤ BMI < 30	30 ≤ BMI < 35	35 ≤ BMI < 40	BMI ≥ 40
N (sex F/M)	162 (152/10)	217 (198/19)	237 (202/35)	328 (262/66)	903 (708/195)	915 (701/214)	893 (779/114)
Age	32.0 ± 1.1 ^a^	32.5 ± 1.0 ^a^	33.4 ± 0.98 ^b^	45.1 ± 0.8 ^c^	48.5 ± 0.5 ^d^	45.9 ± 0.5 ^c^	45.1 ± 0.5 ^c^
Weight (kg)	40.3 ± 0.4	46.7 ± 0.3	58.4 ± 0.5	77.9 ± 0.5	89.7 ± 0.3	103.3 ± 0.4	117.0 ± 0.4
Height (m)	1.65 ± 0.00	1.64 ± 0.01	1.65 ± 0.00	1.66 ± 0.00	1.65 ± 0.00	1.66 ± 0.00	1.62 ± 0.00
BMI (kg/m^2^)	14.67 ± 0.09 ^a^	17.17 ± 0.05 ^b^	21.26 ± 0.12 ^c^	28.03 ± 0.08 ^d^	32.69 ± 0.05 ^e^	37.44 ± 0.05 ^f^	44.25 ± 0.12 ^g^
FM by DXA (kg)	4.4 ± 0.3 ^a^	8.1 ± 0.2 ^a.b^	15.8 ± 0.3 ^b^	30.7 ± 0.3 ^c^	39.6 ± 0.1 ^d^	47.2 ± 0.2 ^e^	57.5 ± 0.2 ^f^
FM by BIA (kg)	7.0 ± 0.3 ^a^ *	9.0 ± 0.1 ^a^ *	13.3 ± 0.2 ^a^ *	25.0 ± 0.3 ^b^ *	34.1 ± 0.1 ^c^ *	43.2 ± 0.2 ^d^ *	56.9 ± 0.2 ^e^ *
Difference of FM (kg)	-2.5 ± 0.2 ^a^	-0.8 ± 0.2 ^a^	2.5 ± 0.2 ^b^	5.6 ± 0.2 ^c^	5.4 ± 0.1 ^c^	4.0 ± 0.1 ^d^	0.6 ± 0.1 ^d^
95% LOA	-9.1; 3.9	-7.3; 5.6	-4.4; 9.5	-2.0; 13.3	-2.6; 13.5	-4.7; 12.7	-9.9; 11.2
FFM by DXA (kg)	35.8 ± 0.3 ^a^	38.2 ± 0.3 ^a^	41.7 ± 0.4 ^b^	45.7 ± 0.4 ^c^	47.7 ± 0.3 ^d^	51.7 ± 0.3 ^e^	54.1 ± 0.2 ^f^
FFM by BIA (kg)	33.5 ± 0.4 ^a^ *	37.6 ± 0.4 ^a^ *	45.1 ± 0.4 ^b^ *	52.9 ± 0.5 ^c^ *	55.5 ± 0.3 ^d^ *	60.0 ± 0.3 ^e^ *	60.0 ± 0.3 ^e^ *
Difference of FFM (kg)	2.2 ± 0.2 ^a^	0.6 ± 0.2 ^a^	-3.3 ± 0.2 ^b^	-7.1 ± 0.2 ^c^	-7.7 ± 0.1 ^c^	-8.2 ± 0.1 ^c^	-5.8 ± 0.1 ^d^
95% LOA	-4.5; 9.0	-5.8; 7.1	-11.3; 4.5	-15.5; 1.1	-16.9; 1.4	-18.6; 2.1	-16.6; 4.8

Values are expressed as means ± sem.

Values without a common letter (a, b, c, d, e, f or g) differ significantly (comparison between BMI groups), p<0.05.

*, p<0.001 vs DXA.

BIA, Bioelectrical Impedancemetry; BMI, body mass index; DXA, Dual X-ray Absorptiometry; FFM, fat-free mass; FM, fat mass; LOA, limits of agreement.

**Fig 1:**
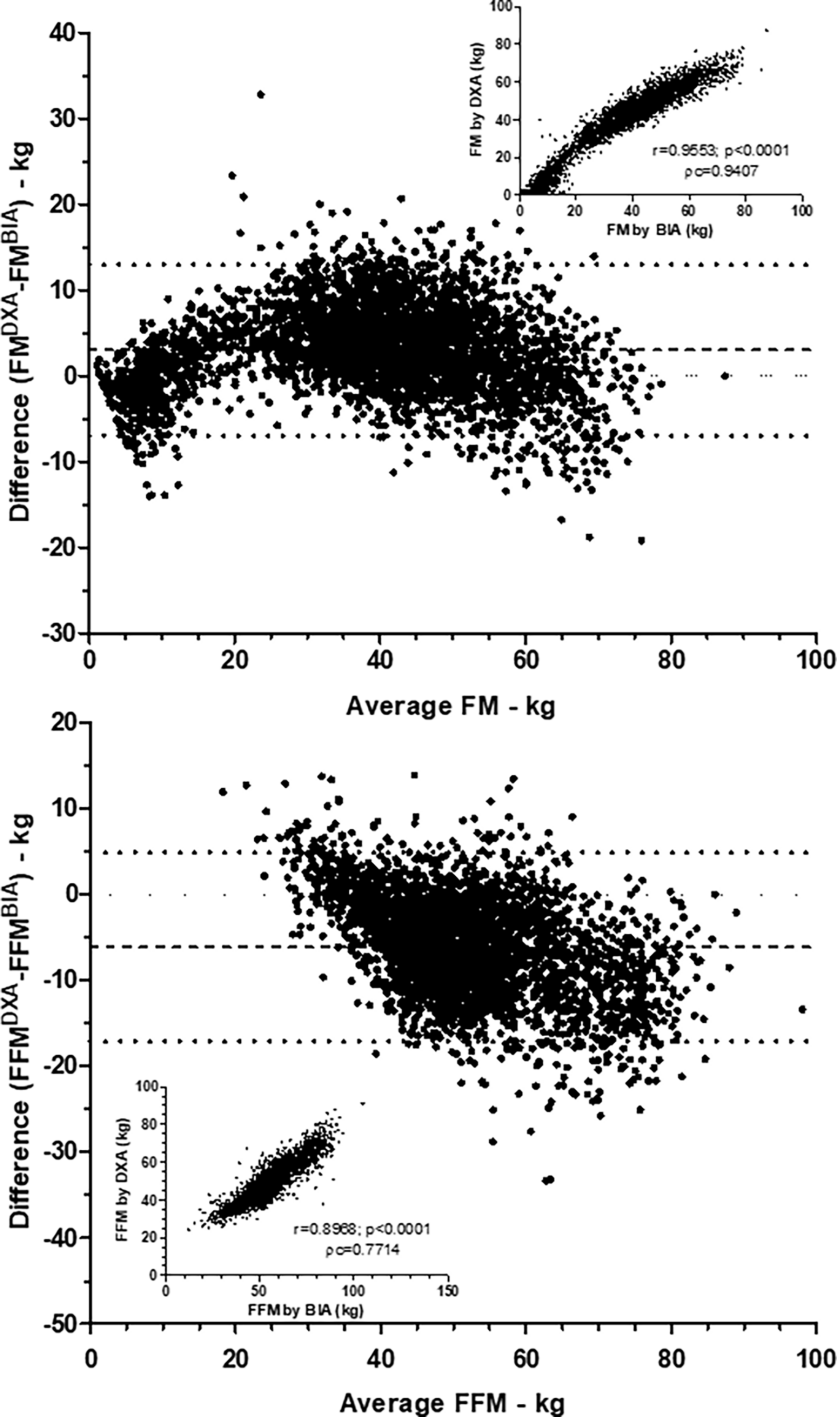
Comparison of fat mass and fat-free mass measurements by DXA and BIA. Fat mass (FM) and fat-free mass (FFM) were measured by DXA (Lunar Prodigy Advance) and BIA (BodyStat Quadscan 4000). Bland Altman plots were created with difference between DXA and BIA for FM and FFM and average of both values. Correlations between values of DXA and BIA were also performed and showed in insert graphs with Pearson r and the concordance coefficient of Lin (ρc).

Taking into account the whole population, whatever the BMI, we observed that values of FM obtained by BIA and DXA were strongly correlated (r = 0.95, p<0.0001, Fig 1, Pearson correlation) with a concordance coefficient of Lin at 0.9407, which is considered as very good [30]. Similar Pearson correlation was observed for FFM (r = 0.89, p<0.0001, Fig 1) while the concordance coefficient of Lin can only be considered as satisfactory (ρc = 0.7714). Bland-Altman plots revealed that differences of FM and FFM obtained by DXA and BIA changed according to the average (Fig 1). In addition, difference of FM between DXA and BIA methods seemed to be negative for extreme values and positive for others. By contrast, difference of FFM between DXA and BIA began positive for the lowest values of FFM to become negative for the highest values of FFM (Fig 1).

Differences in the measurement of FM and FFM by DXA and BIA methods are presented in *Table 1.* For patients with BMI between 16 and 18,5, body composition values measured by DXA and BIA were very closed (difference < 1kg). For BMI ≥ 18,5 and BMI < 40, BIA overestimated FFM from 3.38 to 8.28 kg, and underestimated FM from 2.51 to 5.67 kg compared with DXA method. For BMI < 16, BIA underestimated FFM by 2.25 kg, and overestimated FM by 2.7 kg. DXA and BIA measures were very closed (difference < 1kg) for FM estimation in patients with BMI ≥ 40, while BIA overestimated FFM by 5.87 kg. Interestingly, limits of agreement (LOA) were very large, irrespective of BMI, either for FM and FFM values, as reported in *Table 1*.

**Fig 2:**
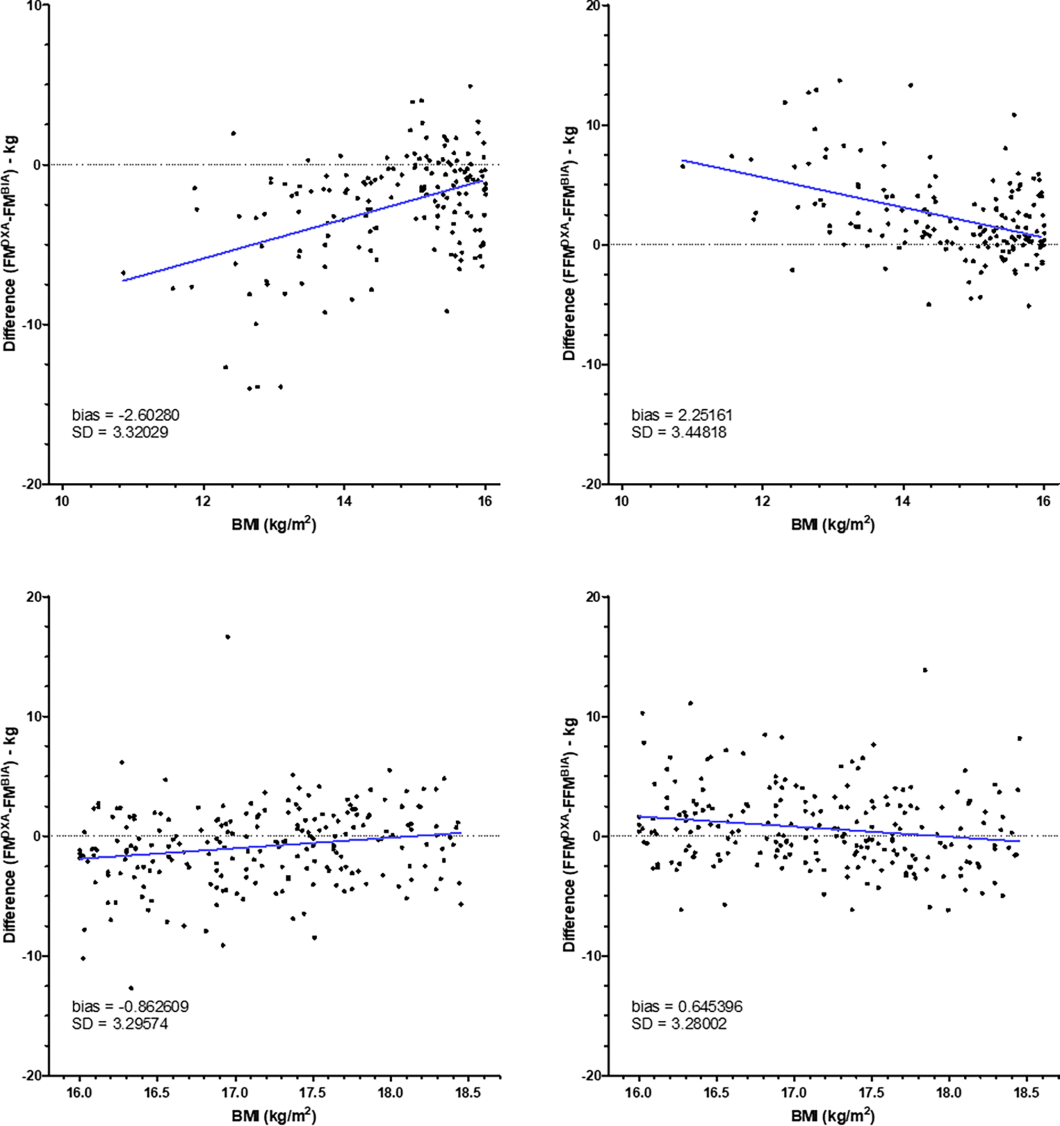
Differences between fat mass and fat-free mass measurements by DXA and BIA in patients with low BMI. Fat mass (FM) and fat-free mass (FFM) were measured by DXA (Lunar Prodigy Advance) and BIA (BodyStat Quadscan 4000) in patients with BMI < 16 kg.m^-2^ (n = 162, upper panels) and patients with BMI between 16 and 18.5 kg.m^-2^ (n = 217, lower panels). Differences of values obtained by DXA and BIA were compared according to the BMI. The blue line represents the linear regression.

**Fig 3:**
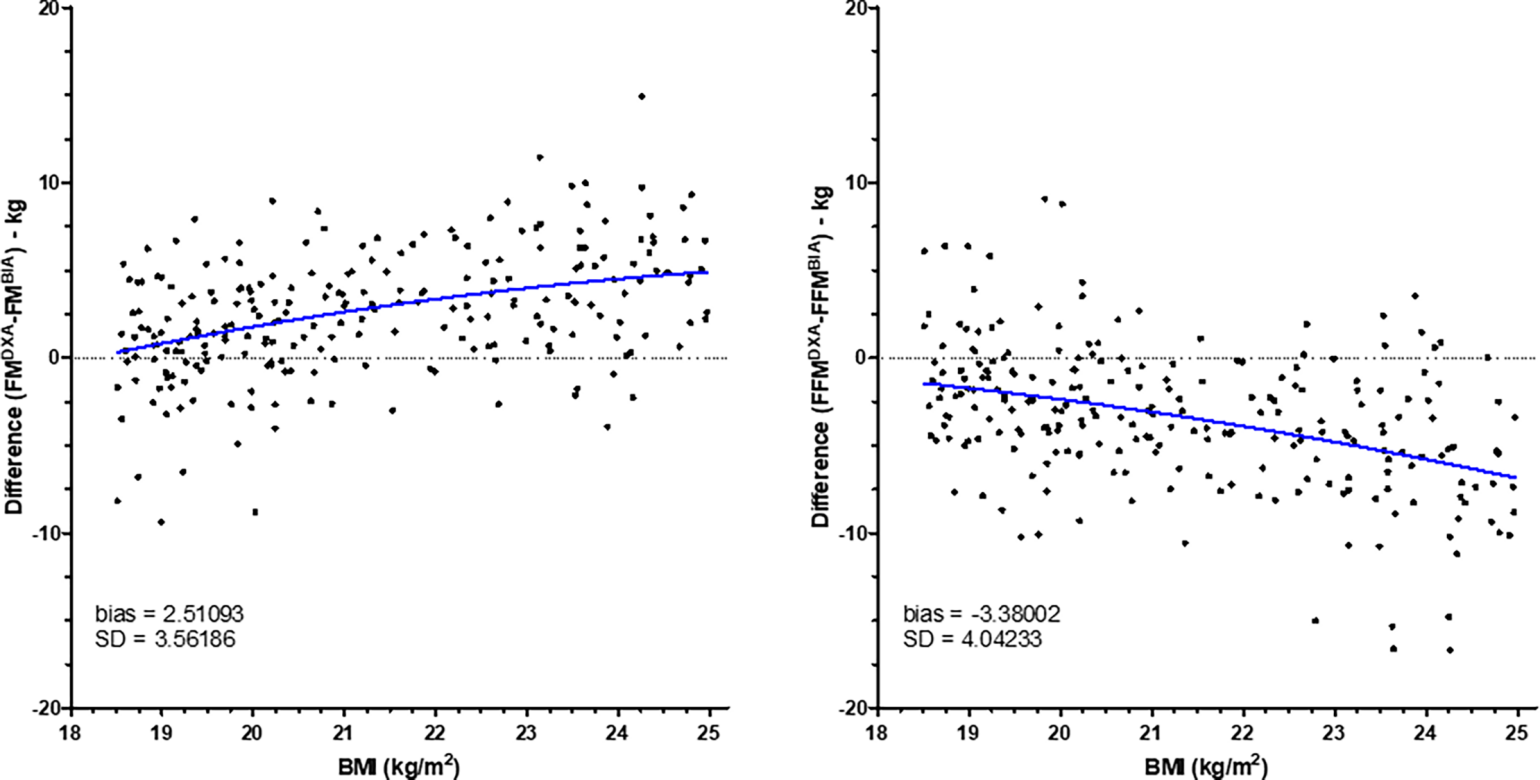
Differences between fat mass and fat-free mass measurements by DXA and BIA in patients with normal BMI. Fat mass (FM) and fat-free mass (FFM) were measured by DXA (Lunar Prodigy Advance) and BIA (BodyStat Quadscan 4000) in patients with BMI between 18.5 and 25 kg.m^-2^ (n = 237). Differences of values obtained by DXA and BIA were compared according to the BMI. The blue line represents the linear regression.

**Fig 4:**
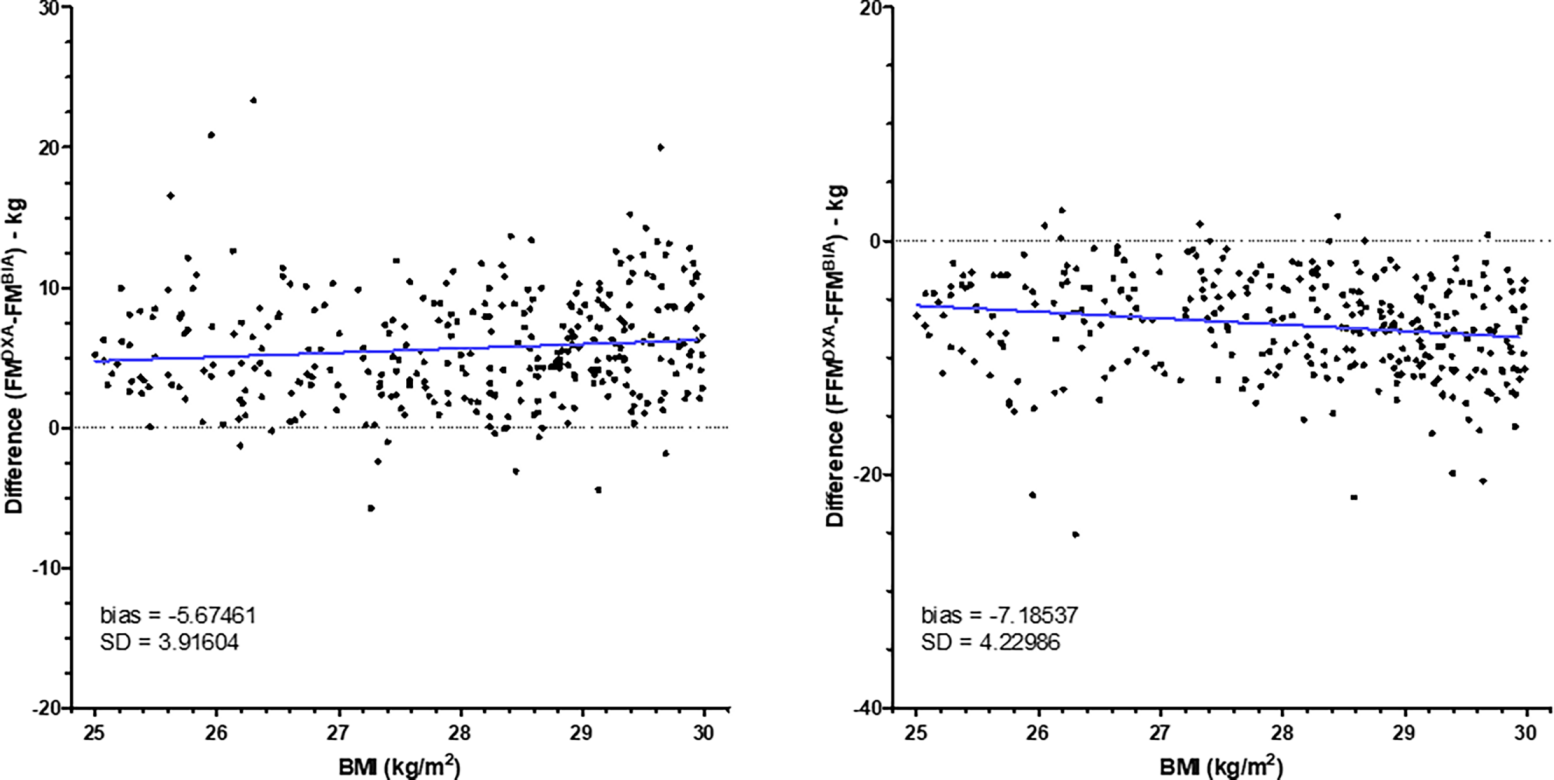
Differences between fat mass and fat-free mass measurements by DXA and BIA in overweight patients. Fat mass (FM) and fat-free mass (FFM) were measured by DXA (Lunar Prodigy Advance) and BIA (BodyStat Quadscan 4000) in patients with BMI between 25 and 30 kg.m^-2^ (n = 328). Differences of values obtained by DXA and BIA were compared according to the BMI. The blue line represents the linear regression.

**Fig 5:**
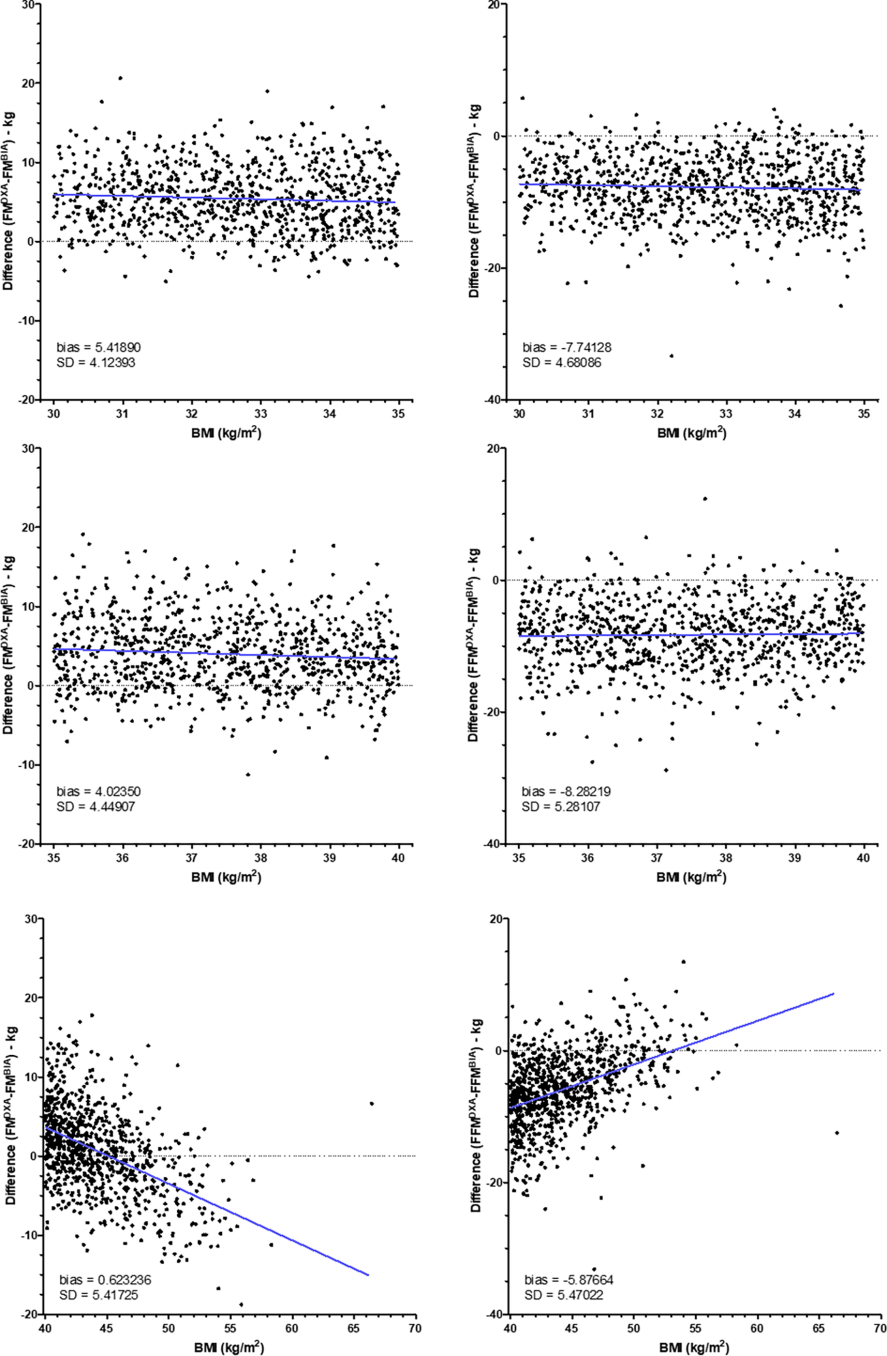
Differences between fat mass and fat-free mass measurements by DXA and BIA in obese patients. Fat mass (FM) and fat-free mass (FFM) were measured by DXA (Lunar Prodigy Advance) and BIA (BodyStat Quadscan 4000) in patients with obesity grade 1 (n = 903, upper panels), grade 2 (n = 915, middle panels) or grade 3 (n = 893, upper panels). Differences of values obtained by DXA and BIA were compared according to the BMI. The blue line represents the linear regression.

However, to know whether differences between BIA and DXA change within each class of BMI, we have created graphs representing differences between BIA and DXA according to the BMI (Figs 2–5). As shown in Fig 2, for patients with BMI < 16, differences for FM and FFM varied with BMI. By contrast, for patients with BMI between 16 and 18.5 (Fig 2), difference did not change. For patients with normal BMI, difference between DXA and BIA for both FM and FFM increased with the increase of BMI (Fig 3). For patients with overweight and grade I and II obesity, difference did not change with BMI (Figs 4 and 5). By contrast, for BMI ≥ 40, differences varied with BMI (Fig 5).

## Discussion

In this present study, we reported small bias, particularly in patients with BMI between 16 and 18, suggesting that BIA and DXA methods are interchangeable at a population level. However, concordance between the two methods at the individual level is lacking, irrespective of BMI.

In patients with BMI between 16 and 18, DXA and BIA measures of FM and FFM were very closed (difference < 1kg). However, LOA ranged from -7.3 to 5.6 kg which is not acceptable in clinical practice. Interestingly, in our population, this BMI class corresponds mainly to patients with anorexia nervosa (AN). This eating disorder is characterized by underweight, protein-energy malnutrition and intense fear of gaining weight [31]. The assessment of body composition plays a key role in evaluating nutritional status in AN, either before or during nutritional rehabilitation [32]. Moreover, a strong correlation between DXA and Computed Tomography was recently found in premenopausal women with AN, whatever hydration level [33]. However, DXA is not routinely used in clinical practice, contrary to BIA [13, 34] whereas no widely disease-specific equation has been accepted for the estimation of body composition in these AN patients. Recently, Marra et al. assessed the accuracy of selected BIA equations [35, 36] for FFM estimation in 82 female patients with AN and reported that all predictive equations underestimated FFM, while the percentage of accurate predictions varied from 12.2% to 35.4% [25]. Interestingly, in this recent study, predictive formulas based on body weight and BIA parameters such as RI (resistance index) and ZI100 (impedance index at 100 kHz) offered a rather accurate prediction of FFM (with high resistance squared) than that observed with anthropometric characteristics only. Thus, authors suggested that performing BIA at frequencies > 50 kHz may be useful in assessing body composition in AN because allowing a more appropriate evaluation of intracellular water [37–39].

Moreover, few previous studies have compared body composition assessment by BIA and DXA methods in AN [35, 36], usually using one specific BIA equation provided by manufacturer. Recently, in order to identify the most suitable BIA equation for 50 AN patients, Mattar et al. compared FM and FFM assessment by DXA and BIA using 5 BIA equations previously validated in healthy population [26]. In this study, the most accurate estimation of FFM and FM was obtained with Deurenberg equation [40] when compared to DXA. Interestingly, no correlation was found between BMI and the differences of measurements of FFM by DXA and BIA methods. Inversely, in our study, we observed that for BMI < 16, differences vary with BMI. In accordance with this result, Piccoli *et al*. reported previously that BIA should not be used in anorexic patients with a BMI <15 because of a lack of accuracy in this BMI class [41]. Few studies have examined the limitations of BIA in underweight patients with AN [34]. The relatively small sample of patients with BMI < 16 in our population studied represents a limitation (162 of 3655); further analyzes are needed in a larger population of patients with BMI < 16.

Furthermore, it seems also relevant to note that in our study, patients with BMI < 18.5 are younger than patients with BMI ≥ 18.5 (p<0.05). In young adults, DXA method reported high levels of accuracy in the measurement of body composition compared with other methods [23, 42, 43]. Moreover, ESPEN (European Society for Parenteral and Enteral Nutrition) recommends to use population-specific equations or equations that adjust FFM and FM changes with age because BIA equations developed in young subjects could lead to large bias in older subjects [13].

Then, we found that for overweight and obese patients (25 < BMI < 40), BIA overestimated FFM from 7.18 to 8.28 kg, and underestimated FM from 4.02 to 5.67 kg compared with DXA method. Again, the LOA were large for this BMI range. This is in accordance with recent results reporting that MF-BIA underestimated FM and overestimated FFM in overweight and obese postpartum women, compared with DXA [16]. Previously, Bosaeus *et al*. also found underestimation of FM by MF-BIA in overweight and obese women, compared to quantitative resonance method [17]. In overweight and obese men (BMI, 28 to 43), Pateyjohns et al. also reported that MF-BIA underestimated FM from 1.06 to 14.25 kg and overestimated FFM from 0.83 to 15.12 kg compared to DXA [21]. Moreover, Panotopoulos *et al*. compared body composition assessment in obese women by three methods: DXA, BIA and NIR spectroscopy, and raised some limits on the use of BIA and NIR to evaluate body composition in clinical research and practice in obese population [22]. Furthermore, BIA also underestimated truncal adiposity in obese women (BMI, 30.4 ± 2.9) compared to DXA [24], and interestingly, differences between these two methods increased with the degree of adiposity. This is in accordance with a recent study showing that underestimation of FM by BIA increased in men with >24.6% body fat and women with >32% body fat, in 403 healthy young adults (BMI, 24 ± 2,8) [23], suggesting that the accuracy of BIA is negatively affected by adiposity as previously reported in overweight women using SF-BIA [44]. Interestingly, Shafer *et al*. explained the underestimation of % body fat in obese subjects by inaccurate estimation of trunk resistance with MF-BIA devices [45]. Thus, further analyzes are needed to evaluate the effect of truncal adiposity and body fat distribution on the accuracy of BIA measurements. Furthermore, limitations of BIA in overweight and obese patients may also be explained by inadequate BIA equations developed in normal-weight subjects, and also by hydration variability [14, 15]. However, few studies reported that BIA accurately estimated TBW in overweight and obese subjects [18, 46]. Surprisingly, in our study, DXA and BIA measures were very closed (difference < 1kg) for FM estimation in patients with BMI ≥ 40, while BIA overestimated FFM by 5.87 kg. Few previous studies also reported good concordance between the two methods in overweight and obese subjects [19, 20]. Nevertheless, in our study, we have seen that in patients with BMI ≥ 40, differences between DXA and BIA varied with BMI.

### Strengths and limitations

Although many studies have previously compare measurement of body composition by DXA and BIA, to our knowledge, this is the largest retrospective study which allows comparison of these devices according to BMI ranges, in adult outpatients followed in a Nutrition Unit. A first limitation of our study is that patients were mainly women (82% of the total population). Sex differences in measurement of body composition by DXA and BIA have been poorly studied. However, recent data reported no effect of sex on TBW measurement by BIA method in hemodialysis patients [47] and in healthy subjects [46]. Secondly, we used only one MF-BIA device (BIA, Bodystat Quadscan 4000) whose proprietary equation is unknown and probably not adapted to each BMI class of patients.

In conclusion, our study reported the lack of concordance between BIA and DXA methods at the individual level, irrespective of BMI. Future studies are needed in order to develop new BIA specific equations according to the BMI class.
